# Complete Genome Sequence of *Bacillus subtilis* Strain CGMCC 12426, an Efficient Poly-γ-Glutamate Producer

**DOI:** 10.1128/genomeA.01163-17

**Published:** 2017-10-26

**Authors:** Hui Dong, Jinglin Chang, Xin He, Qinlian Hou, Wei Long

**Affiliations:** Key Laboratory of Tianjin Radiation and Molecular Nuclear Medicine, Institute of Radiation Medicine, Chinese Academy of Medical Sciences & Peking Union Medical College, Tianjin, China

## Abstract

*Bacillus subtilis* CGMCC 12426 is an efficient producer of poly-γ-glutamate with regular stereochemistry. Here, the complete genome sequence of *B. subtilis* CGMCC 12426 is presented, which may facilitate the design of rational strategies for further strain improvements with industrial potential.

## GENOME ANNOUNCEMENT

Poly-γ-glutamate (γ-PGA) is an anionic, biodegradable, water-soluble biopolymer that is edible and nontoxic to humans and the environment (1). This biopolymer has various functions and has been used in a broad range of industrial fields such as food, cosmetics, pharmaceuticals, and water treatment (2, 3). γ-PGA was first discovered in *Bacillus anthracis* at the start of the 20th century, and γ-d-PGA was later produced by this type of strain (4). Several bacteria (mostly from the genus *Bacillus*) have been shown to secrete γ-PGA into the medium as a product of fermentation. The most intensively studied are *B. subtilis* and *B. licheniformis* (5, 6). Compared with *B. licheniformis*, *B. subtilis* shows a higher productivity, and there has been growing interest in the application of this type of strain (7, 8). Recently, a strain of *B. subtilis* was isolated from soil and named *B. subtilis* KH2, which is an efficient γ-PGA producer. It has been deposited in the China General Microbiological Culture Collection Center (CGMCC no. 12426).

Genomic DNA from *B. subtilis* CGMCC 12426 was extracted using the QIAamp DNA minikit (Qiagen, CA). The quantity and quality of genomic DNA were evaluated on the Agilent 2100 Bioanalyzer (Agilent, USA). Genomic DNA was used to construct a 10-kb insert SMRTbell library, and then sequenced on the single molecule real-time (SMRT) DNA sequencing platform using the Pacific Biosciences (PacBio) RS II sequencer (Pacific Biosciences, CA) (9). A total of 150,292 polymerase reads on one SMRT cell for 3-h movie times led to a total of 1,386,478,854 nucleotide bases. After filtering to remove any reads having low accuracy values less than 0.8, 1,256,996,185 read bases were obtained with 0.866 read quality. All of the filtered sequences were *de novo* assembled using the RS hierarchical genome assembly process (HGAP) assembly protocol 2.0 in SMRT analysis software version 2.3.0 (Pacific Biosciences) (10). The length of the complete circular chromosome is 4,138,265 bp, with a 74,165-bp length plasmid. The annotation was performed by the NCBI Prokaryotic Genome Annotation Pipeline (PGAP), resulting in the prediction of 4,581 genes, including 4,222 coding sequences (CDSs), and 87 tRNA and 30 rRNA (5S rRNA, 16S rRNA, and 23S rRNA) sequences.

The genome sequence of *B. subtilis* CGMCC 12426 could serve as a basis for further elucidation of the genetic background of this promising strain, and provide significant opportunities for investigating the metabolic and regulatory mechanisms underlying the formation of ethanol, organic acids, amino acids, etc. Importantly, all of the genes responsible for PGA biosynthesis and degradation were successfully annotated. This genome sequence may also facilitate the identification of suitable target genes that can assist with the development of superior microbial cell factories with higher concentration, yield, and productivity of PGA by systems metabolic engineering.

### Accession number(s).

The complete genome information of *B. subtilis* KH2 (CGMCC 12426) was deposited in GenBank under the accession numbers CP018184 and CP018185.
